# Experimental Verification of Theoretical Stress-Strain Model for Compressed Concrete Considering Post-Peak Stage

**DOI:** 10.3390/ma15176064

**Published:** 2022-09-01

**Authors:** Iakov Iskhakov, Ilya Frolov, Yuri Ribakov

**Affiliations:** Department of Civil Engineering, Ariel University, Ariel 40700, Israel

**Keywords:** experimental study, compressed concrete, theoretical stress-strain model, ascending and descending branches

## Abstract

The theoretical stress-strain model for compressed composite cement materials’ behavior without empirical coefficients was proposed by Iskhakov in 2018. This model includes the following main parameters describing concrete behavior: stresses and strains corresponding to the border between the elastic and non-elastic behavior stages of a concrete specimen, ultimate elastic strains, and stresses and strains at the end of the post-peak region. Particular attention is focused on the descending branch of the stress-strain diagram, as well as on the analysis of concrete elastic and plastic potentials. These potentials are important for assessing the dynamic response of the concrete element section, as well as for concrete creep analysis. The present research is aimed at experimental verification of the above-mentioned theoretical model. The obtained experimental results are in good agreement with the theoretical ones, which confirms the model’s accuracy and enables a significant reduction in the empirical coefficients number in compressed reinforced concrete elements design. This, in turn, represents the scientific novelty of this study.

## 1. Introduction

The stress-strain relationship is the fundamental feature of concrete response under external loads in the entire range of its behavior. Currently, available models describing this relationship are mainly based on experimental data. This approach is also reflected in modern design codes [1,2].

One of the first attempts to investigate the compressed concrete stress-strain ($\sigma_{c}-\varepsilon_{c}$) relationship was conducted at the beginning of the previous century [3]. Later, Hognestad investigated 190.5 cm-long compressed concrete column specimens with square sections of 25.4 × 25.4 cm and 223.5 cm long ones with circular sections 30.5 cm in diameter [4]. An analytical parabolic model was proposed to describe the ascending branch of the diagram, and the descending branch was represented by a linear dependence. This model was later modified by using new experimental data obtained for concrete specimens with a height-to-diameter ratio of 2 subjected to compressive loading controlled by a constant deformation rate [5,6]. However, the modified model is described by a more complicated mathematical expression. In the authors’ opinion, it is a general problem of empirical relationships that leads to the questionable accuracy of models and does not have theoretical confirmation.

The empirical relationship between $\sigma_{c}$ and $\varepsilon_{c}$ for compressed concrete, presented earlier [5], was further investigated [7,8]. Modified versions of the empirical coefficient were proposed to consider the stress-strain diagram shape [7] and the changes in concrete modulus of elasticity [8]. Additionally, the descending branch curve of this diagram was also determined by these models [7,8]. Additionally, the model, which has experimental and theoretical significance, was proposed for simplifying the procedure, assuming parameters of a section, such as low axial strains, transverse deformations, and elastic behavior. Besides that, this method is an approximated one and can be easily implemented [9]. Analysis of available theoretical and experimental studies shows that the problem of using empirical coefficients to describe concrete behavior remains relevant in recent years [10,11,12].

According to the modern design codes [1,2], the $\sigma_{c}-\varepsilon_{c}$ relationship for normal strength compressed concrete classes up to C60 is a diagram that includes a linear part in the elastic zone and a convex parabolic curve in the non-elastic one. Following those codes, ultimate elastic–plastic deformations of compressed concrete are equal to 2‰, and considering the descending branch—3.5‰—the concrete elastic behavior stage is limited by 40% of the strength.

An important issue is obtaining the descending branch of the compressed concrete $\sigma_{c}-\varepsilon_{c}$ diagram because this branch, together with the ascending one, determines the potential of concrete in compression, which is essential for dynamic loads [13]. It should be noted that when analyzing concrete response in the descending branch range, some researchers consider the modulus of elasticity as a negative value [14]. In our opinion, the algebraic sign of the modulus, in this case, is not important and only emphasizes a significant reduction in concrete stresses. However, the exact stage up to which the mentioned stress reduction occurs is more important. The definite answer to this question is given in RILEM recommendations [15], stating that the stresses decrease to half the concrete’s load-bearing capacity, i.e., to $0.5\;f_{c}$. The present study is also focused on this problem.

Another issue related to the descending branch investigation is considering the joint work of a testing machine and a compressed specimen. The influence of a testing machine’s insufficient stiffness on the obtained deformations in the compressed concrete specimen was identified [16,17]. As a result, using a closed-loop testing machine controlled by specimen strain feedback was proposed for obtaining a full stress-strain diagram for compressed concrete [15,18,19]. Some other requirements for test specimens were formulated based on available experimental results [15,20]:

– Provide a certain ratio of specimen height to its cross-section size;
– Take into account the friction between a specimen and planes of a testing machine and reduce this influence as far as possible;
– A minimum of three longitudinal external sensors should be placed around a specimen between testing machine plates;
– The loading rate should correspond to the static loading (about 0.1 μm/s).

The present research is devoted to in-depth experimental investigations of the above-mentioned problems, including new methods for their implementation. In particular, this investigation is based on the previous theoretical results and on generalizing the $\sigma_{c}-\varepsilon_{c}$ model [13], as well as the Mini-Max Principle [21], the Quasi-isotropy concept of the RC element at the ultimate limit state (ULS) [22], and the Structural Phenomenon [23].

## 2. Research Aims and Novelty

The main objectives of the study are:

– Experimental verification of the theoretical stress-strain model for compressed concrete;
– Analysis of the model post-peak stage and comparison of experimental results with theoretical ones.

The novelty of this study is in experimental justification of the above-mentioned theoretical model and its following parameters:

– Exact border between elastic and inelastic concrete behavior;
– Ultimate elastic deformation and elastic potential of compressed specimen section;
– Limit stress of the $\sigma_{c}-\varepsilon_{c}$ diagram descending branch.

The results of this study provide a basis for updating the above-mentioned parameters.

## 3. Short Description of the Theoretical Stress–Strain Model

The theoretical stress-strain model for compressed concrete is based on the following three known principles: the Mini-Max Principle [21], Quasi-isotropy concept of RC element at ULS [22], and the Structural Phenomenon [23]. Each of these principles provides an additional equation (or system of equations) for determining RC element parameters under uniaxial compression, compression with eccentricity, and bending at ULS [21]. This enables the design of such elements without additional empirical coefficients. The summary of each of these three principles is presented below.

The Mini-Max Principle idea is determined by obtaining the real load-bearing capacity of the structure, avoiding under- or over-estimation [21]. This becomes possible by selecting the compressed zone section depth (static parameter), which corresponds to the simultaneous implementation of the structure’s maximum load-bearing capacity when the external load value acting on the failure zone (kinematic parameter) reaches its minimum. As a result, the Mini-Max Principle provides a solution for the unity theorem of the limit equilibrium method by combining the static and kinematic approaches in the same calculation, which means using both extreme features of failure load. At the same time, only one method is used in calculations according to the limit equilibrium method—kinematic or static.

The essence of the Quasi-isotropy idea is that a bent reinforced concrete element section at ULS is calculated assuming that it is made of a non-linear isotropic material [22]. This approach enables the obtainment of an effective section with a minimum reinforcement ratio while the compressed zone depth reaches its maximum value, equal to half of the effective section depth [24]. As a result, an additional equilibrium equation is obtained. This excludes the need for empirical coefficients, similar to the case when the Mini-Max Principle was applied.

The Structural Phenomenon was proposed as a result of extensive experimental and theoretical data analysis of reinforced concrete structures’ behavior at three levels: the material level and levels of static and dynamic loadings [23]. The phenomenon’s essence is that parameters of material and structure at ULS increase or decrease twice compared to the values in the elastic state. This phenomenon was proved for symmetrical sections of structural elements subjected to symmetrical loading. Using the Structural Phenomenon, as well as the above-described principles, provides additional equations that also eliminates the empirical coefficient.

It can be summarized that using the above-described principles allows to obtain theoretical solutions to many new and existing problems, which could be previously solved by using empirical coefficients. Thus, further development of the limit equilibrium method and the general theory of reinforced concrete elements became possible.

**Figure 1:**
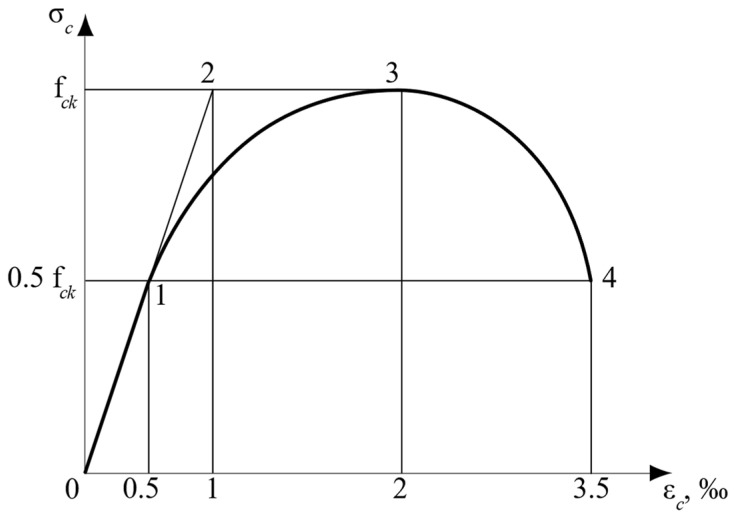
Theoretical compressed concrete stress-strain diagram, following [13,25]: 1. The end of concrete section elastic behavior; 2. section elastic potential under dynamic loading and creep; 3. the initial point of descending branch, which has the same shape as the ascending one; 4. the point determining the full section potential.

The three above-described principles enabled the formulation of the main provisions for the theoretical model of the compressed concrete stress-strain relationship (Figure 1) [13]. The novelty of this model was the theoretical approach for finding the stress-strain diagram parameters without applying the empirical coefficients. The main parameters of this diagram for concrete classes up to C50 are:

– The maximum value of elastic deformations $\varepsilon_{c\;el}=0.5‰$;
– The limit concrete elastic potential determined by the deformation $\varepsilon_{c\;el\;ul}=1‰$ (this potential is generally required for structural analysis under dynamic loads and for concrete creep analysis) [25];
– The ultimate elastic stress reaching half of the concrete strength, i.e., $\sigma_{c\;el}=0.5f_{ck}$;
– The boundary of the diagram descending branch is determined by the stress equal to $\sigma_{c\;ul}=0.5f_{ck}$ (helps find the real value of the compressed concrete cross-section potential).

## 4. Experimental Program

### 4.1. Selecting the Specimens

In the present study, six cylindrical specimens with 15 cm diameter and 30 cm height subjected to axial loading were tested in order to obtain the experimental stress-strain relationship for normal-strength concrete, including the descending branch. The research included two test series with three specimens in each of them, marked as Sp1.1, Sp1.2, Sp1.3 and Sp2.1, Sp2.2, and Sp2.3, respectively. The concrete strength, corresponding to C20/25 and C35/45 concrete classes, was determined using three 15 × 15 × 15 cm cubic specimens for each test series, respectively [1,26]. The lower-strength concrete was chosen to obtain more pronounced specimen behavior in the nonelastic stage, including the descending branch.

Cylindrical specimens were chosen to avoid local stress concentration problem, which is pronounced, for example, in the corners of the prismatic ones [20]. Thus, the edge effect was eliminated. The ratio of 2 between the specimen diameter and height (15:30 cm) ensures an unrestrained area in the middle of the specimen’s height [20], which is especially important for obtaining the descending branch.

### 4.2. Material Properties

Ready-mix concrete and concrete produced in laboratory conditions were used in the first and second test series. Concrete mixture for the first series was prepared using the following materials:

– Locally produced composite 52.5N CEM II/AM-SLV Portland cement with portions of granulated blast furnace slag (S) and siliceous fly ash (V) from 18 to 30% [27];
– Water-reducing and retarding admixture (G100X) with a density of 1.212 kg/dm^3^;
– Retarding and water-reducing admixture (HGP) with a density of 1.188 kg/dm^3^;
– Mix of three different types of locally available quarry sand with a fraction size of 0–1.2 mm, 0–2.5 mm, and 0–5 mm;
– Locally available quarry crushed dolomite limestone with a coarse aggregate fraction of 9–16 mm according to [28].
– The materials for preparing the second test series’ concrete mixture were:
– Locally produced composite 42.5N CEM II/B-LL Portland cement with portions of limestone between 21 and 35% [27];
– Locally available quarry sand with a fraction size of 0–4.75 mm;
– Locally available quarry crushed dolomite limestone with a coarse aggregate fraction of 19/14 mm according to [28].

A 0.045 m^3^ rotating pan mixer was used for concrete mixture preparation, and the calculated mixture volume was 0.043 m^3^.

The final concrete mixture compositions for dry aggregates are presented in Table 1. The composition used for the second test series was as per the previous authors’ study [29].

The concrete mixture was cast into steel molds and compacted by tamping and rodding in 2 and 3 layers for cubic and cylindrical specimens, respectively, following [30,31]. All specimens were stored for 24 h after casting, covered by a polyethylene film [32]. After demolding, specimens were cured for 27 days in a fog room under a constant temperature of 23±2 °C and relative humidity of 95±5%. The compressive strength was obtained at 28 days, according to [1,33].

### 4.3. Test Setup

A servo-hydraulic closed-loop class 1 testing machine (with a maximum error of 1% FS) and a maximum load of 3000 kN was used to carry out concrete cylinders under axial compression [20]. The rigidity of the test frame corresponds to normative requirements [34] for excluding the influence of testing machine properties on post-peak concrete behavior records. The tests were conducted under a displacement control mode at a constant loading rate of 0.01 mm/min. Control signal was obtained by a sensor measuring loading head displacement (PressLDT). The data obtained from this sensor were additionally used for the specimen post-peak behavior analysis that will be described below. An external pressure sensor was installed in the testing machine oil line to measure the applied load.

The following measurement equipment was used to obtain the compressed concrete stress-strain experimental relationship. Eight strain-gauges (SG) with 30 and 60 mm bases were used in the specimen longitudinal direction to investigate the behavior in the ascending branch range. SGs were placed in pairs (one 30 mm and one 60 mm SG in pairs at 90 degrees intervals) to duplicate the obtained experimental results. Both sensor types had a resolution of up to 0.1 micro-strains ($10^{-6}$), a resistance of 120 Ohm, and a gauge factor of 2.09 and 2.08 for 30 and 60 mm SGs, respectively. The 60 mm SG length was chosen according to the requirement of exceeding the coarse-aggregate maximum dimensions by at least three times [20].

Two circumferential 60 mm-long SGs were used in the transverse direction. Additionally, two longitudinal highly accurate transducers (HAT) with a 50 mm measurement base, sensitivity of 0.02 microns, and a ±1.5 mm stroke were attached to the specimen’s surface in the transverse SGs location zone. Thus, twelve sensors were used for testing each specimen. All sensors were placed in the specimen central zone, which is considered to be uninfluenced by boundary conditions, i.e., friction forces between the testing machine plates and specimen surfaces.

**Figure 2:**
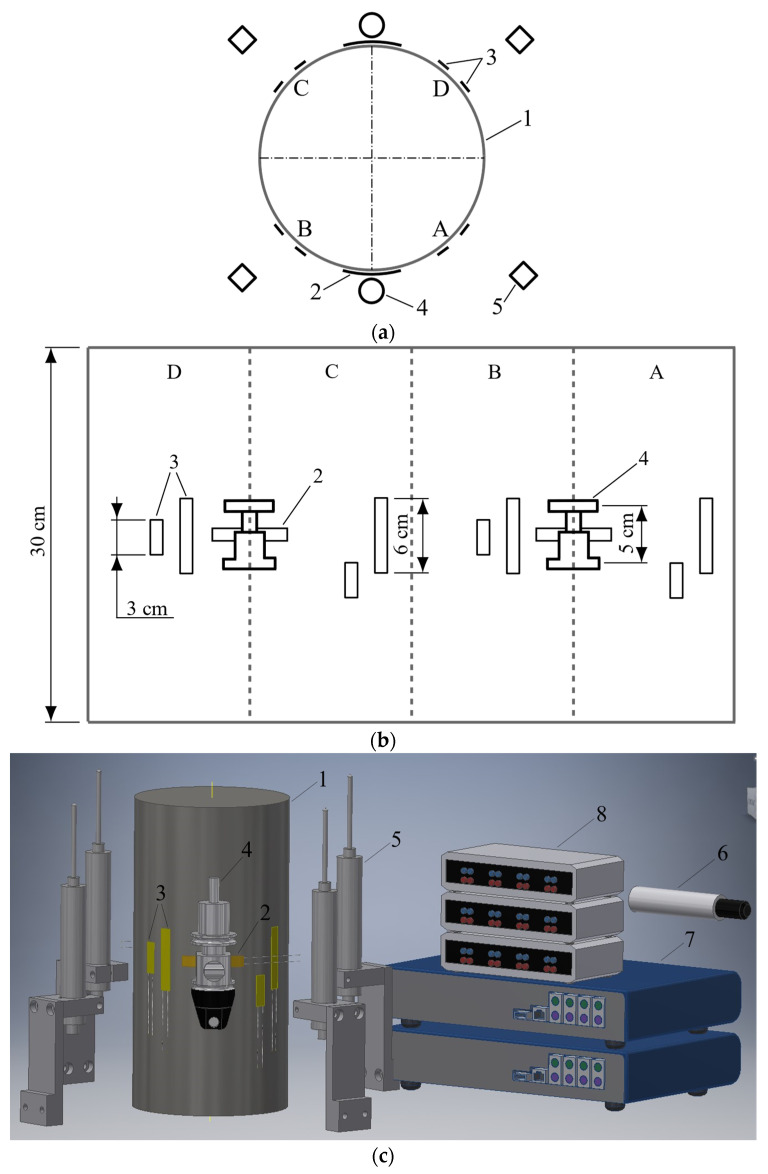

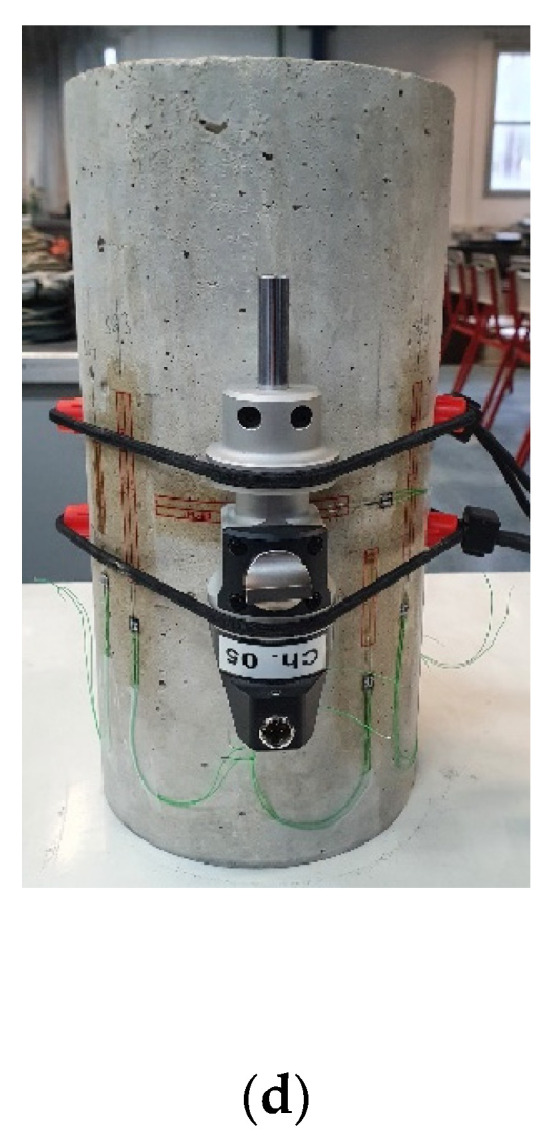
Testing setup: (**a**) Measurement device location scheme; (**b**) Specimen surface development; (**c**) General view; (**d**) Specimen before loading. 1. Cylindrical concrete specimen; 2. Transverse SG sensors; 3. Longitudinal SG sensors; 4. HAT devices; 5. LDT devices; 6. Load sensor; 7. Data logger; 8. Wheatstone bridge; A, B, C, and D sectors (according to Figure 2a).

Four linear displacement transducers (LDT) with a 50 mm stroke and a high resolution (<1 μm) were mounted between the testing machine loading plates in order to obtain accurate response of a specimen in the post-peak stage (descending branch). A general view of the test setup is shown in Figure 2.

The upper surface of the cylindrical specimens was leveled by a thin layer of sifted fine-grain sand to obtain an additional effect of reducing the friction between the testing machine plate and a specimen.

## 5. Experimental Results and Discussion

### 5.1. Data Processing Methodology

Experimental data processing included three stages. In the first stage, data obtained from the longitudinal SGs and from the HAT sensors were analyzed. These data allowed us to obtain the ascending branch of the compressed concrete $\sigma_{c}-\varepsilon_{c}$ diagram. In the second stage, data from sensors attached to the testing machine (PressLDT and LDTs) were evaluated (see Figure 2a). These data were used to obtain the descending branch of the $\sigma_{c}-\varepsilon_{c}$ diagram. In the third stage, the curves obtained in the first and second stages were combined into a complete stress-strain relationship of compressed concrete specimens.

### 5.2. Analysis of the Experimental $\sigma_{c}-\varepsilon_{c}$ Concrete Diagrams

**Figure 3:**
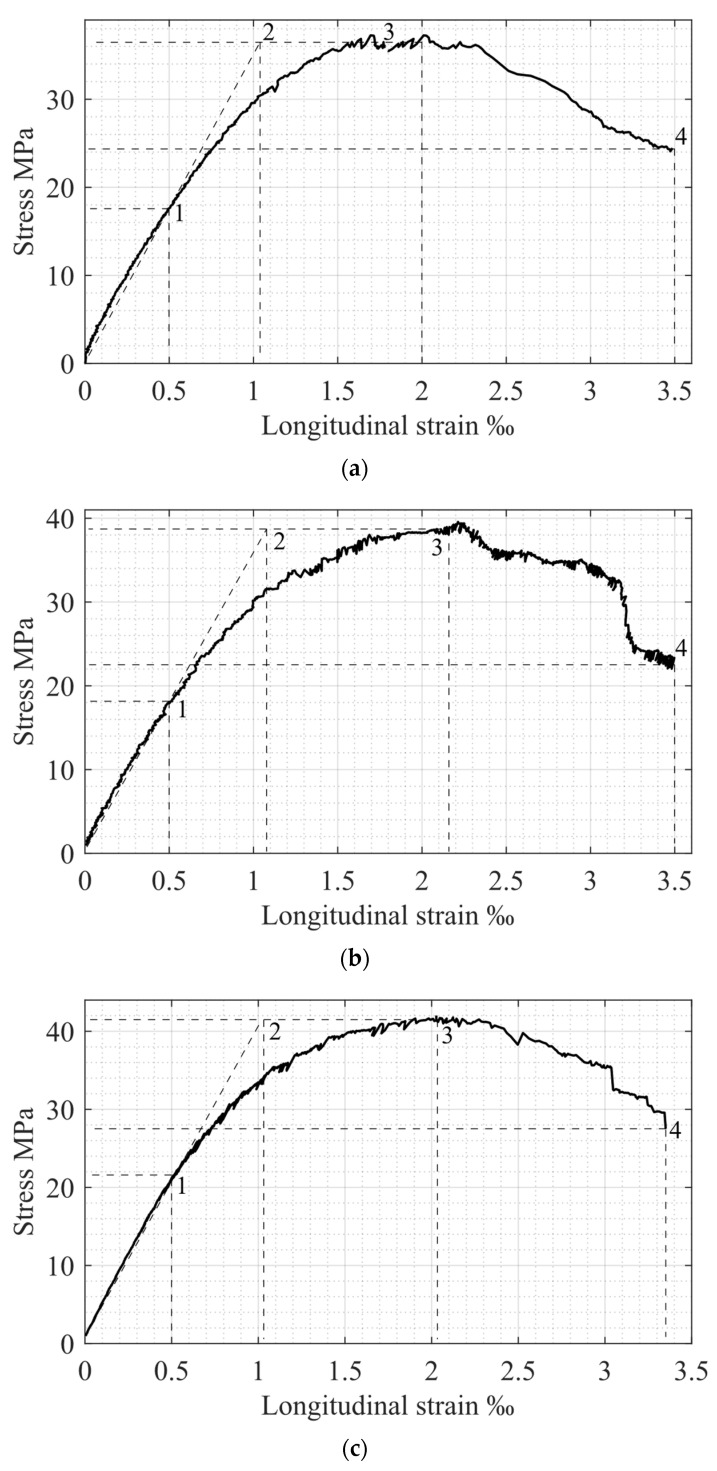

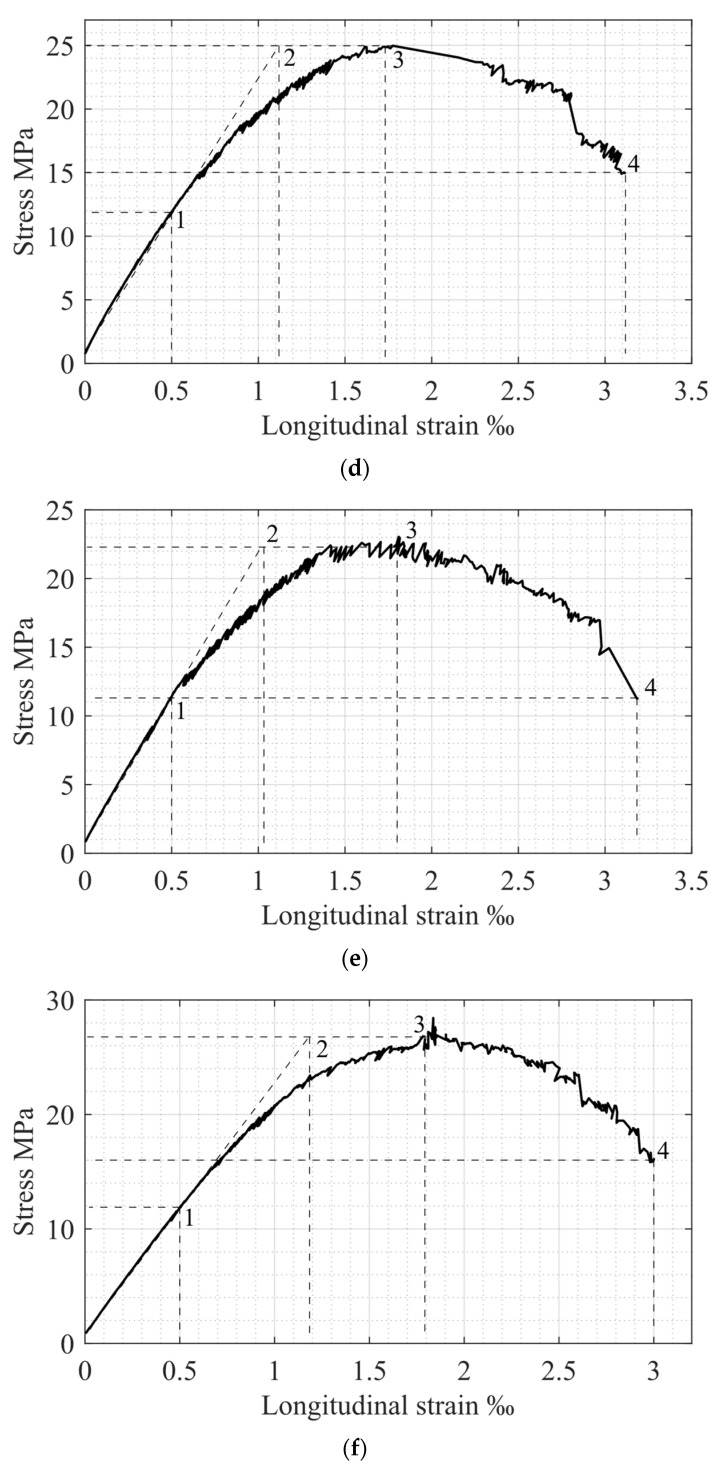
Experimental relationships $\sigma_{c}-\varepsilon_{c}$: (**a**) Specimen Sp1.1; (**b**) Specimen Sp1.2; (**c**) Specimen Sp1.3; (**d**) Specimen Sp2.1; (**e**) Specimen Sp2.2; (**f**) Specimen Sp2.3.

The elastic and elastic–plastic behavior of compressed specimens are determined by maximum and ultimate elastic strains, $\varepsilon_{c\;el}$ and $\varepsilon_{c\;el\;ul}$ (see points 1 and 2 in Figure 1). The specified deformations correspond to stresses $\sigma_{c\;el}$ and $f_{ck}$, respectively. The post-peak concrete behavior stage is characterized by peak and ultimate stresses and strains (see points 3 and 4 in Figure 1). The corresponding experimental $\sigma_{c}-\varepsilon_{c}$ relationships are presented in Figure 3. The obtained experimental values for the main $\sigma_{c}-\varepsilon_{c}$ diagram parameters, as well as relations between strains, strength, and stresses in elastic and ultimate stages, are shown in Table 2 and Table 3.

The post-peak concrete behavior area is characterized by the descending branch of the $\sigma_{c}-\varepsilon_{c}$ diagram between points 3 and 4 in Figure 1. It is determined by stresses $\sigma_{c\;ul}=0.5f_{ck}$ and strains $\varepsilon_{c\;ul}\approx 3.5‰$. Experimental results for these parameters are shown in Table 3.

The analysis of variation coefficients (CV), based on standard deviations, for the experimental data presented in Table 2 and Table 3 shows sufficient convergence of the obtained results (Table 4). The maximum CV value is 14.5%, which is acceptable for concrete considering its natural features, e.g., material heterogeneity, as well as possible load-application eccentricities, etc.

**Figure 4:**
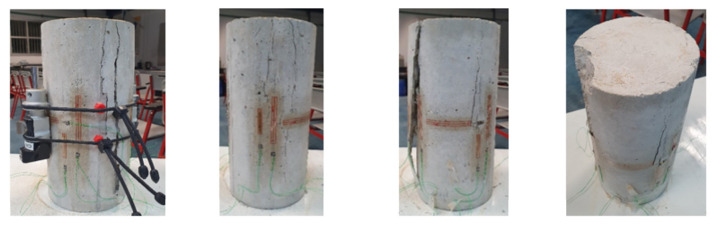
Typical specimen-failure schemes.

Typical failure schemes of experimental specimens are presented in Figure 4.

## 6. Comparison of Experimental and Theoretical Results

**Figure 5:**
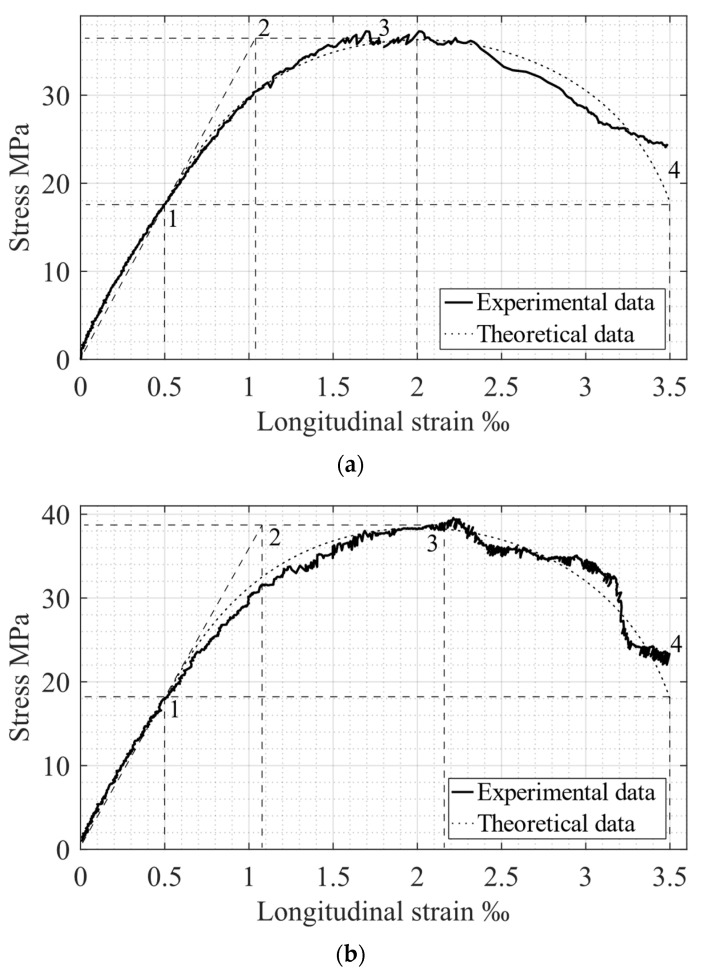

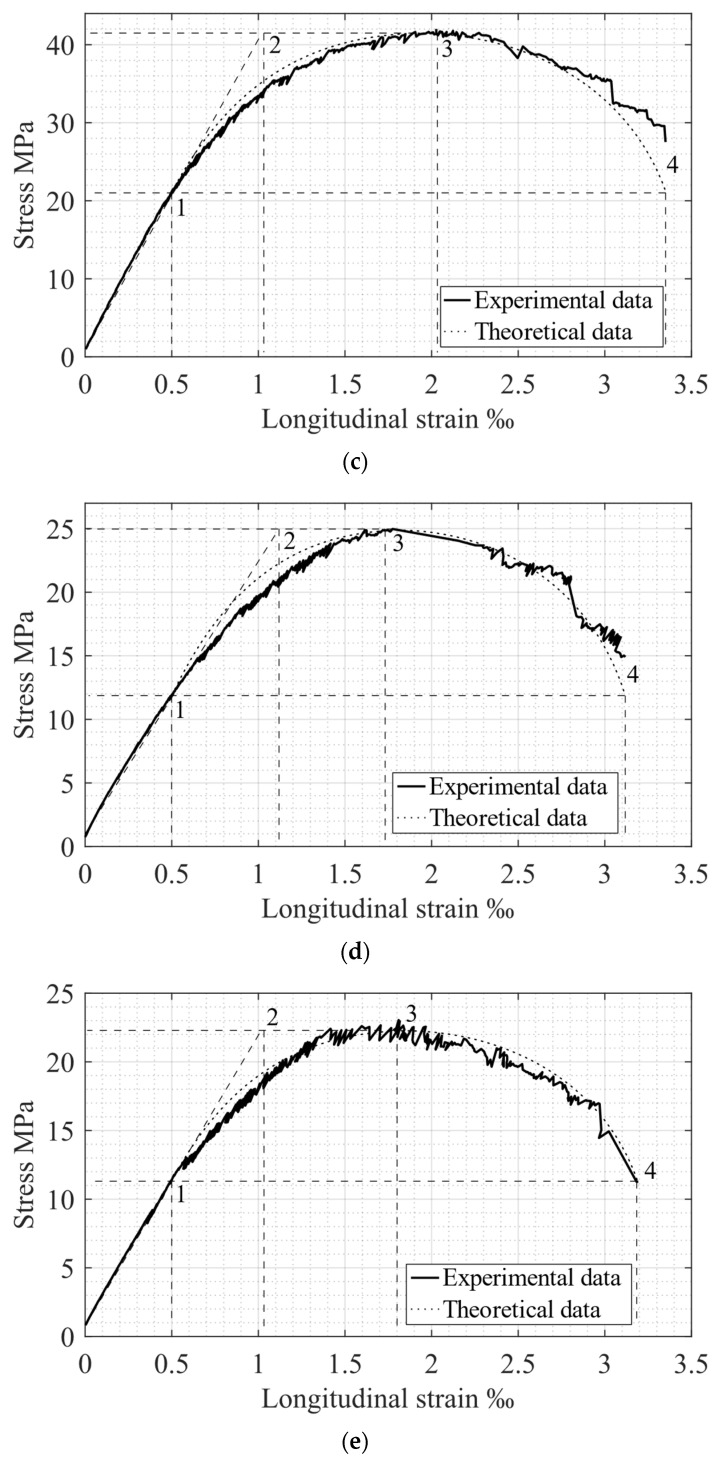

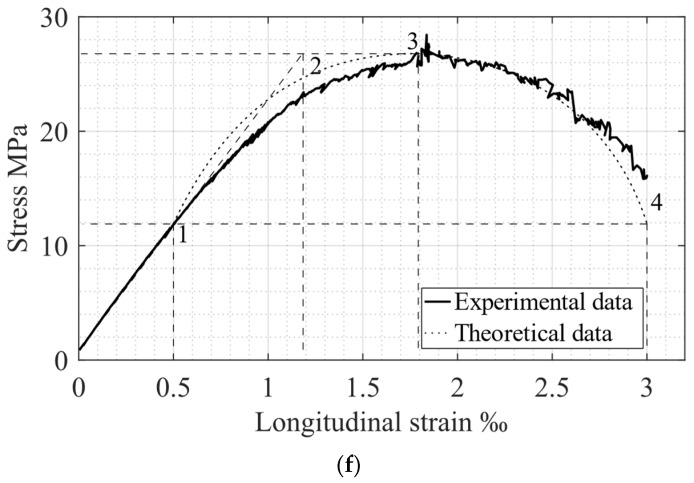
Comparison of experimental and theoretical $\sigma_{c}-\varepsilon_{c}$ relationships: (**a**) Specimen Sp1.1; (**b**) Specimen Sp1.2; (**c**) Specimen Sp1.3; (**d**) Specimen Sp2.1; (**e**) Specimen Sp2.2; (**f**) Specimen Sp2.3.

As mentioned above, the main parameters for concrete behavior under compressive load are stresses and strains, determined by points 1–4 in Figure 1 and Figure 3. A comparison of the corresponding experimental and theoretical data is presented in Table 5. Consequently, a graphical representation of theoretical and experimental $\sigma_{c}-\varepsilon_{c}$ diagrams is shown in Figure 5. The results of comparing the theoretical and experimental potentials values of the stress-strain diagrams [13] are presented in Table 6.

The analysis of the data presented in Table 5 shows rather good convergence. A comparison of elastic and plastic potentials, presented in Table 6, also demonstrates good agreement between theoretical and experimental results. Further comparison of the relation between $U_{el}^{Exper}$ and $U_{pl}^{Exper}$ confirms the Structural Phenomenon concept [23]: the elastic parameters increase twice when specimens reach the plastic stage. Taking everything into account, the entire range of the obtained experimental data and its comparison with the theoretical one completely confirms the proposed theoretical stress-strain model for compressed concrete.

## 7. Conclusions

The experimental results fully confirm the following main parameters of the theoretical stress-strain model for compressed concrete:

– Stresses and strains define the border between the elastic and inelastic compressed concrete behavior;
– The elastic potential of compressed concrete is important for dynamic analysis and investigations of reinforced concrete structure creep.

In the frame of the present research, geometrical correspondence between the descending branch in the compressed concrete stress-strain diagram and the ascending one was verified. It was also confirmed that the experimentally-obtained value of the descending branch limit stress is $0.5f_{ck}$, which corresponds to the theoretical prediction.

The present experimental investigations fully confirm the theoretical stress-strain diagram of compressed concrete. As a result, this diagram can be called the “Law of Concrete” and included in the modern concrete theory. This law ensures the proper and accurate design of compressed reinforced concrete elements without empirical coefficients, which is important for the further development of structural concrete theory.

## Figures and Tables

**Table 1:** Concrete mixture proportioning.

Components	Units	Series 1	Series 2
Cement, C	kg/m^3^	275	448.4
Water, W	kg/m^3^	158	224.2
Water–cement ratio, W/C	-	0.57	0.50
Sand, S	kg/m^3^	914	602.2
Crushed stone, CS	kg/m^3^	942	1017.8
Water-reducing and retarding admixture, WR	% of C	1.6	-
Retarding and water-reducing admixture, RW	% of C	0.1	-
$\mathrm{Fresh}\;\mathrm{concrete}\;\mathrm{density}\;\mathrm{in}\;\mathrm{compacted}\;\mathrm{state},\;\rho_{c}$	kg/m^3^	2293	2293

**Table 2:** Experimental values of the $\sigma_{c}-\varepsilon_{c}$ diagram ascending branch parameters.

Scheme	$\varepsilon_{c\;el}$, ‰	$\varepsilon_{c\;el\;ul}$, ‰	$\varepsilon_{c1},\;‰$	$\frac{\varepsilon_{c\;el}}{\varepsilon_{c\;el\;ul}}$	$\sigma_{c\;el},\;\mathrm{MPa}$	$f_{ck},\;\mathrm{MPa}$	$\frac{\sigma_{c\;el}}{f_{ck}}$
Sp1.1	0.50	1.03	2.00	0.49	17.50	36.50	0.48
Sp1.2	0.50	1.06	2.16	0.47	18.10	38.70	0.47
Sp1.3	0.50	1.03	2.03	0.49	20.99	41.60	0.50
Average	0.50	1.04	2.06	0.48	18.86	38.93	0.48
Sp2.1	0.50	1.12	1.72	0.45	11.92	24.94	0.48
Sp2.2	0.50	1.06	1.80	0.47	11.35	22.35	0.51
Sp2.3	0.50	1.19	1.79	0.42	11.86	26.40	0.45
Average	0.50	1.12	1.77	0.45	11.71	24.56	0.48

**Table 3:** Experimental values of the $\sigma_{c}-\varepsilon_{c}$ diagram descending branch parameters.

Specimen	$\varepsilon_{c\;ul},\;‰$	$\sigma_{c\;ul},\;\mathrm{MPa}$	$\frac{\sigma_{c\;ul}}{f_{ck}}$
Sp1.1	3.50	24.10	0.66
Sp1.2	3.50	22.40	0.58
Sp1.3	3.35	27.53	0.66
Average	3.45	24.67	0.63
Sp2.1	3.12	14.91	0.60
Sp2.2	3.19	11.27	0.50
Sp2.3	3.00	16.05	0.61
Average	3.10	14.08	0.57

**Table 4:** Variation coefficients for the $\sigma_{c}-\varepsilon_{c}$-diagram-controlled parameters.

	Test Series	$\varepsilon_{c\;el}$	$\varepsilon_{c\;el\;ul}$	$\varepsilon_{c1}$	$\frac{\varepsilon_{c\;el}}{\varepsilon_{c\;el\;ul}}$	$\frac{\sigma_{c\;el}}{f_{ck}}$	$\varepsilon_{c\;ul}$	$\sigma_{c\;ul}$	$\frac{\sigma_{c\;ul}}{f_{ck}}$
CV, %	1	0.00	1.36	3.37	1.35	3.18	2.05	8.65	6.12
	2	0.00	4.73	2.01	4.72	5.00	2.53	14.50	8.21

**Table 5:** Comparison of average experimental and theoretical values of the main $\sigma_{c}-\varepsilon_{c}$ diagram parameters.

Data		$\varepsilon_{c\;el}$	$\varepsilon_{c\;el\;ul}$	$\varepsilon_{c1}$	$\frac{\varepsilon_{c\;el}}{\varepsilon_{c\;el\;ul}}$	$\frac{\sigma_{c\;el}}{f_{ck}}$	$\varepsilon_{c\;ul}$	$\frac{\sigma_{c\;ul}}{f_{ck}}$
Theoretical		0.50	1.00	2.00	0.50	0.50	3.50	0.50
Average experimental	Series 1	0.50	1.04	2.06	0.48	0.48	3.45	0.63
	Series 2	0.50	1.12	1.77	0.45	0.48	3.10	0.57

**Table 6:** Experimental and theoretical values of elastic and plastic potentials and relation between them.

Specimen	$U_{el}^{Theor}$, kPa	$U_{el}^{Exper}$, kPa	$U_{pl}^{Theor},\;\mathrm{kPa}$	$U_{pl}^{Exper},\;\mathrm{kPa}$	$\frac{U_{el}^{Exper}}{U_{pl}^{Exper}}$	$\mathrm{Average}\;\frac{U_{el}^{Exper}}{U_{pl}^{Exper}}$
Sp1.1	31.94	32.87	63.88	65.81	0.50	0.53
Sp1.2	33.86	38.72	67.73	63.13	0.61	
Sp1.3	36.40	36.69	72.80	71.95	0.51	
Sp2.1	21.82	18.87	43.65	39.54	0.48	
Sp2.2	19.56	17.14	39.11	37.54	0.46	
Sp2.3	23.10	21.72	46.20	37.07	0.59	

## Data Availability

Not applicable.
